# Plasma Lipidomic Profiles in cART-Treated Adolescents with Perinatally Acquired HIV Compared to Matched Controls

**DOI:** 10.3390/v16040580

**Published:** 2024-04-09

**Authors:** Julie van der Post, Thiara E. J. Guerra, Malon van den Hof, Frédéric M. Vaz, Dasja Pajkrt, Jason G. van Genderen

**Affiliations:** 1 Department of Pediatric Infectious Diseases, Amsterdam UMC, Location Academic Medical Center, University of Amsterdam, 1100 DD Amsterdam, The Netherlands; 2 Amsterdam Reproduction and Development Research Institute, Amsterdam, The Netherlands; 3 Department of Epidemiology and Data Science, Amsterdam UMC, Location Academic Medical Center, University of Amsterdam, 1105 AZ Amsterdam, The Netherlands; 4 Amsterdam Public Health, Ageing & Later Life, Health Behaviors and Chronic Diseases, Amsterdam, The Netherlands; 5 Department of Clinical Chemistry and Pediatrics, Laboratory Genetic Metabolic Diseases, Emma Children’s Hospital, Amsterdam UMC, Location Academic Medical Center, University of Amsterdam, Meibergdreef 9, 1105 AZ Amsterdam, The Netherlands; f.m.vaz@amsterdamumc.nl; 6 Amsterdam Gastroenterology Endocrinology Metabolism, Inborn Errors of Metabolism, Amsterdam, The Netherlands; 7 Core Facility Metabolomics, Amsterdam UMC, Location University of Amsterdam, Meibergdreef 9, 1105 AZ Amsterdam, The Netherlands; 8 Amsterdam Infectious Diseases and Immunology Research Institute, Amsterdam, The Netherlands

**Keywords:** human immunodeficiency virus, cardiovascular disease, combination antiretroviral therapy, lipidomics, lipid profiles

## Abstract

Children with perinatally acquired human immunodeficiency virus (PHIV) are growing into adulthood with HIV and treatment-associated comorbidities, such as dyslipidemia and insulin resistance. HIV is identified as independent risk factor for cardiovascular disease (CVD). The hypothesis behind increased CVD risk associated with HIV includes vascular inflammation, dyslipidemia and combination antiretroviral therapy (cART) metabolomic toxicity. To investigate differences in lipid profiles and pathophysiological mechanisms of CVD risk in adolescents with PHIV, we compared the plasma lipidome of PHIV adolescents and HIV-negative controls. We additionally investigated the influence of current cART regimens and increased lipoprotein(a) (Lp(a)) levels on the plasma lipidome. We included 20 PHIV-infected adolescents and 20 HIV-negative controls matched for age, sex, ethnic origin and socio-economic status. Plasma lipidome was measured using Thermo Scientific Ultimate 3000 binary high-performance liquid chromatography (HPLC)–mass spectrometry. We evaluated the plasma lipidome in PHIV adolescents using different cART regimens (including those known to be associated with lipid alterations). The median age was 17.5 years (15.5–20.7) and 16.5 years (15.7–19.8) for PHIV adolescents and controls, respectively. Of PHIV adolescents, 45% used a non-nucleotide reverse transcriptase inhibitor (NNRTI)-based (25%) or protease inhibitor (PI)-based (20%) cART regimen. In this pilot study, we observed no significant differences between lipidomic profiles between PHIV adolescents and controls. We observed no differences in the plasma lipidome in participants with increased versus normal Lp(a) levels. Different cART regimens appear to influence chain length differences in the plasma lipidome of PHIV adolescents; however, the significance and causality of this observation remains undetermined. Further research on the influence of cART on lipid composition could further identify these alterations.

## 1. Introduction

With the introduction of combination antiretroviral therapy (cART), the incidence of severe HIV- or AIDS-related complications has declined and survival rates of children and adolescents with perinatally acquired HIV (PHIV) have significantly improved [1]. Despite adequate viral suppression with cART, long-term complications such as increased cardiovascular risk (CVD) and metabolic syndrome (MetS) are still eminent in HIV-infected individuals [2,3]. Furthermore, HIV is considered an independent risk factor for the development of CVD in HIV-infected adults, with lifelong treatment increasing this risk [3,4]. In PHIV children with adequate viral suppression, HIV-associated CVD manifestations include cardiac abnormalities, endothelial dysfunction, subclinical vascular disease, increased carotid intima-media thickness, increased metabolic risk and insulin resistance [4,5,6,7,8]. Previous studies suggest an increased CVD risk and dyslipidemia with alterations in levels of low-density lipoprotein cholesterol (LDL-C), high-density lipoprotein cholesterol (HDL-c), triglycerides (TG) or lipoprotein(a) (Lp(a)) in PHIV children and adolescents compared to healthy controls [9,10,11,12]. Lp(a) is a lipoprotein responsible for cholesterol transport in blood and its levels are predominantly influenced by genetic predisposition, and fluctuations can vary depending on the ethnic background [13,14]. Elevated levels of Lp(a) have been associated with CVD risk in the general population [15]. In PHIV children and adolescents, knowledge on Lp(a) dynamics is scarce, but high baseline Lp(a) levels have been reported in PHIV children and adolescents, suggesting increased CVD risk while growing up [9,16].

The exact pathophysiological mechanisms underlying HIV-associated CVD risk are yet to be elucidated and hypotheses include ongoing HIV-related vascular inflammation and cART-related metabolic toxicity [17,18,19]. Treatment with cART has been associated with metabolic alterations such as weight gain, changes in adipose tissue and lipid abnormalities [20]. Specifically, protease inhibitors (PIs) and non-nucleoside reverse transcriptase inhibitors (NNRTIs) were found to increase CVD risk with the development of lipodystrophy, an abnormal fat distribution (including weight gain) or diabetes [21,22]. Today, lipodystrophy is less often seen with recent cART regimens as compared to older regimens but associations between weight gain and the current frequently used integrase inhibitors (INSTIs) have been reported [22]. Typically, cART can be categorized into seven classes based on the mechanism of action, including nucleoside reverse transcriptase inhibitors (NRTIs), NNRTIs, PIs and integrase strand transfer inhibitors (INSTIs) [23]. NRTIs such as tenofovir inhibit the reverse transcriptase enzyme vital for HIV replication and NNRTIs such as efavirenz inhibit reverse transcriptase by binding to an alternative site that causes confirmational change which disrupts the function of the enzyme. PIs such as darunavir target the protease enzyme, halting the maturation of new virus particles [23]. Approved integrase inhibitors such as dolutegravir disrupt viral integration, while long-acting therapies like bictegravir enhance the future efficacy of HIV treatment. In cART treatment regimens, commonly, a backbone of two NRTIs is combined with a third class, such as an NNRTI, INSTI or PI, to form the therapeutic approach [24]. Although lipids (including Lp(a)) play a key role in the pathophysiology of CVD risk, the currently used biomarkers may inadequately depict changes in lipid metabolism [25,26,27]. The progress in plasma lipidomic studies has proven its potential in comprehending the mechanisms of increased CVD risk, prediction, treatment strategies and therapy monitoring in the general population [28,29]. Lipidomics investigates lipid composition, structure and function within biological systems, employing advanced analytical techniques to elucidate the possible effects on or role in health and disease risk. Moreover, it is suggested that it has surpassed traditional lipid biomarkers in CVD risk assessment and prediction [30,31]. For instance, in HIV-infected adults, associations have been observed between metabolic syndrome, CVD risk and cardiovascular events and several lipid classes and/or species such as diacylglycerols (DAGs), phosphatidylinositol (PI), phosphatidylglycerol (PG), triacylglycerol/triglycerides (TAGs), specific ceramide (CER), lysophosphatidylethanolamines (LPEs) and cholesterol esters (CEs) [30,31,32]. There is a strong correlation between specific DAG and TAG alterations and future cardiovascular events [31].

Lipidome analyses in PHIV children and adolescents are lacking, and investigations into the lipid concentration and composition of individual lipid species can possibly identify possible risk profiles and disease markers and evaluate metabolic risk. Only one study suggests altered lipidome profiles in PHIV children related to ongoing inflammation, indicative of increased CVD risk [33]. To date, comparisons between lipid profiles and currently used cART regimes for CVD risk calculation are missing. Lipidomic profiling may help to further elucidate the pathophysiological mechanisms between HIV and CVD risk [34].

To further explore CVD risk and its potential mechanisms in PHIV adolescents, the primary objective of the current study is to investigate differences in lipid profiles between PHIV adolescents on cART and matched HIV-negative controls. Additionally, we aim to examine the influence of different cART regimens on the plasma lipidome in PHIV adolescents. Moreover, we seek to assess the impact of elevated levels of Lp(a) on lipid composition.

## 2. Materials and Methods

### 2.1. Study Design and Population

This cross-sectional pilot study was conducted as part of the Neurological, Cognitive and Visual performance in HIV-infected Children (NOVICE) study, a cohort study investigating neurological, cognitive, ophthalmological and cardiovascular impairment in cART-treated PHIV adolescents and compared to healthy controls matched for age, sex, ethnic origin and socio-economic status (SES). SES was defined as the level of parental education and occupational status. Between February 2017 and July 2018, children and adolescents with PHIV visiting the outpatient clinic of the Emma Children’s Hospital, Amsterdam UMC, or those who had previously participated in the NOVICE study were approached and were eligible for inclusion. The study characteristics are previously described in [35]. HIV-negative controls were recruited from similar communities. Exclusion criteria were having non-HIV associated neurological disease, a history of intracerebral neoplasms or infection, severe traumatic brain injury (with loss of consciousness longer than 30 min), or a psychiatric disorder. We adhered to the tenets of the Declaration of Helsinki and obtained informed consent from all parents or legal guardians of participants younger than 16 years and participants aged older than 12 years. The Ethics Committee of the Amsterdam University Medical Center approved the study, which is registered at the Dutch Trial Registry (identifier: NL6813).

### 2.2. Demographics, Disease and Treatment-Related Characteristics

HIV and treatment-related data were identified through patient record data and provided by Sichting HIV Monitoring (SHM, HIV Monitoring Foundation, Amsterdam, The Netherlands). Additional treatment and demographic-related characteristics were collected through questionnaires. An undetectable viral load of HIV-1 RNA was defined as having fewer than 40 copies/mL. HIV-negative status was confirmed in all control participants.

### 2.3. Lipidomics

#### 2.3.1. Sample Collection, Biomarker Analysis and cART Regimens

Non-fasting plasma blood samples were collected through venipuncture from all participants and were stored −80 °C until further analysis. Lp(a) analysis was selected based on the potential causal relation between elevated Lp(a) levels and CVD disease risk [36,37]. Lp(a) levels were assessed using Vitalab Selectra E chemistry analyzer with reagents from Diasys (Diasys, Waterbury, CT, USA), with threshold values set at ≥30 mg/dL as previously described [9]. For the analysis of different cART regimens in PHIV adolescents and plasma lipidomics, the usage of cART was defined as using at least three antiretroviral drugs from a minimum of two drug classes. For each participant, we collected clinical data on exposure to antiretroviral therapy (ART), including current treatment such as NRTIs, NNRTIs, PIs, JNSTIs and other active treatment.

#### 2.3.2. Plasma Lipidomic Profiling

Lipidomic analysis of samples was performed at the Core Facility Metabolomics of the Amsterdam UMC, location AMC, Amsterdam, The Netherlands, using high-performance liquid chromatography (HPLC)–mass spectrometry with two analytical columns in two ionization modes, as previously described [38,39]. In a 2 mL tube, the following amounts of internal standards dissolved in 1:1 (*v*/*v*) methanol/chloroform were added to each sample: diacylglycerol DG(14:0)2 (0.5 nmol), triacylglycerol TG(14:0)3 (0.5 nmol), cholesterol ester D7-CE(16:0) (2.5 nmol), phosphatidylcholine PC(14:0)2 (0.5 nmol), phosphatidylserine PS(14:0)2 (0.02 nmol), phosphatidylethanolamine PE(14:0)2 (0.05 nmol), phosphatidic acid PA(14:0)2 (0.02 nmol), sulfatide SM4(17:0) (0.01 nmol), phosphatidylinositol PI(8:0)2 (0.05 nmol), lysophosphatidylcholine LPC(14:0) (1 nmol), lysophosphatidylethanolamine LPE(14:0) (0.01 nmol), lysophosphatidic acid LPA(14:0) (0.02 nmol), ceramide phosphocholines SM(d18:1/12:0) (0.125 nmol), sphingosine SPH(d17:0) (0.125 nmol), sphingosine SPH(d17:1) (0.125 nmol), ceramide Cer(d18:1/12:0) (0.125 nmol), ceramide Cer(d18:1/25:0) (0.125 nmol), sphingosine-1-phosphate S1P(d17:0) (0.125 nmol), sphingosine-1-phosphate S1P(d17:1) (0.125 nmol), glucosylceramide GlcCer(d18:1/12:0) (0.125 nmol), lactosylceramide LacCer(d18:1/12:0) (0.125 nmol) and ceramide-1-phosphate Cer1P(d18:1/12:0) (0.125 nmol). A total of 1.5 mL of 1:1 (*v*/*v*) chloroform/methanol was added before thorough mixing.

The 20 µL plasma samples were centrifuged at 14.000× *g* rpm for 5 min, after which the supernatant was transferred to a glass vial and evaporated under a stream of nitrogen at 60 °C. The resulting residue was dissolved in 100 μL of a 1:1 (*v*/*v*) mixture of chloroform and methanol. Lipids analysis was conducted using a Thermo Scientific Ultimate 3000 binary HPLC coupled to a Q Exactive Plus Orbitrap mass spectrometer (Thermo Fisher Scientific, Waltham, MA). For normal phase separation, of each sample, 2 μL was injected onto a Phenomenex^®^ LUNA silica (250 mm × 2 mm; 5 µm 100Å). The column temperature was maintained at 25 °C. The mobile phase consisted of (A) 85:15 (*v*/*v*) methanol/water containing 0.0125% formic acid and 3.35 mmol/L ammonia and (B) 97:3 (*v*/*v*) chloroform/methanol containing 0.0125% formic acid. Using a flow rate of 0.3 mL/min, the LC gradient included a dwell at 10% A from 0 to 1 min, followed by a ramp to 20% A at 4 min, a ramp to 85% A at 12 min, a ramp to 100% A at 12.1 min, a dwell at 100% A between 12.1 and 14 min, a ramp to 10% A at 14.1 min and a dwell at 10% A for 14.1–15 min. For reversed-phase separation, 2 μL of each sample was injected onto a Waters HSS T3 column (150 mm × 2.1 mm; 1.8 μm particle size). The column temperature was maintained at 60 °C and the mobile phase comprised solvent A, with a ratio of 4:6 (*v*/*v*) of methanol/water, and solvent B, with a ratio of 1:9 (*v*/*v*) of methanol/isopropanol. Both solvents contained 0.1% formic acid and 10 mmol/L ammonia. With a flow rate of 0.4 mL/min, the LC gradient protocol involved dwelling at 100% A at 0 min, followed by a ramp to 80% A at 1 min, a subsequent ramp to 0% A at 16 min, a dwell period at 0% A for 16–20 min, a ramp to 100% A at 20.1 min, and a final dwell at 100% A for 20.1–21 min. Mass spectrometry data were collected via negative and positive ionization modes by continuous scanning across the range from *m*/*z* 150 to *m*/*z* 2000. Data analysis was performed using a lipidomics pipeline developed in-house, implemented in R programming language (http://www.r-project.org) and MATLAB lipid identification relied on accurate mass determination, relative retention times, analysis of samples with known metabolic abnormalities and injection of relevant standards. In the lipidomics pipeline, lipid classes were defined based on generic chemical formula, where the radyl group is represented by “R”. Upon integration of the lipid database into the annotation pipeline, each lipid class’s generic chemical formula was expanded by substituting the “R” element with various radyl group lengths and levels of unsaturation/double bonds. The resulting comprehensive list of chemical formulas was then employed to compute the neutral monoisotopic mass for each species. This monoisotopic mass was subsequently translated into a series of *m*/*z* values for each adduct/charge combination that could reliably annotate the specific species. Reported lipid abundances are semi-quantitative, determined by dividing the analyte’s response (peak area) by that of the corresponding internal standard, and multiplied by the concentration of the internal standard (arbitrary unit, A.U) as previously visualized in more detail with visual material [40]. The notation “-O” denotes lipids containing an alkyl-ether group, while “-P” signifies an alkenyl-ether group. In cases where the nature of the ether species (alkyl/alkenyl) cannot be ascertained or chromatographically separated, this is denoted by “(O + P)”. We denoted identified phospholipids in our analysis as C(XX:Y), where C denotes the lipid class, XX denotes the total number of carbon atoms and Y the total number of double bonds in the (combined) fatty acyl chains, for example P(41:5). There is no dedicated internal standard for ether lipids available. We therefore used the PC(14:0)2 and PE(14:0)2 to normalize the corresponding ether lipid species as previously described [40].

### 2.4. Statistical Analysis

For demographic participant data, standard descriptive statistics were used and the data were compared using Student’s *t*-test for normally distributed or a Mann–Whitney U test for non-normally distributed continuous data or Fisher’s exact test for categorical data. We conducted a plasma lipidomic analysis with the calculated data of individual lipid species for the lipidomic analysis. The plasma lipidomics analysis was initially performed between PHIV adolescents and controls and furthermore to compare elevated Lp(a) levels to levels of Lp(a) within the reference range and to identify variations in the lipidomic profiles among PHIV adolescents receiving different cART regimens, specifically INSTI-based regimens compared to NNRTI/PI-based regimens. We performed a principal component analysis (PCA) with MixOmics in R for the comparison between these groups, and Student’s *t*-test was used to compare individual lipid species. A *p*-value of 0.05 or less was considered significant. We performed PCA with the variable influence on projection (VIP) score to estimate the importance of variables. A partial least squares (PLS) regression analysis was conducted to evaluate shared factors between the groups together with a discriminant analysis (PLS-DA) to assess potential differences among the groups based on underlying metabolite intensities. Subsequently, VIP scores were computed from the PLS-DA results. VIP scores served to estimate the significance of each variable in the PLS model. Variables with VIP scores close to or exceeding 1 were recognized as significant in the analysis [41]. Heatmaps were generated with the z scores of the individuals from each individual sample in a row by using the following formula Z = (X (value of the individual sample)—µ (average of row)) divided by the standard deviation. Volcano plots were generated to identity differences in lipid species between the two groups, with significance calculated as (−log10(*p*-value)), with a *p*-value of 0.01 or less considered significant, versus effect size (log2(fold change)) on the *y* and *x* axes, respectively.

## 3. Results

### 3.1. Participant Characteristics

The participants’ characteristics are summarized in Table 1. In total, 40 participants were included, of which 20 were PHIV adolescents and 20 were matched controls. The median age was 17.5 years (IQR 15.5–20.7) and 16.5 years (IQR 15.7–19.8) for PHIV adolescents and controls, respectively. We found no significant differences between PHIV participants and controls in age, sex, ethnic origin, systolic and diastolic blood pressure and BMI (each *p* > 0.05). Of the PHIV participants, 18 (90%) had an undetectable viral load. Of the PHIV participants, 11/20 (55%, elvitegravir or dolutegravir) used an INSTI-based regimen. Other PHIV participants currently used an NNRTI- or PI-based regimen (ritonavir, darunavir, efavirenz or rilpivirine), namely, 9/20 (45%).

### 3.2. Lipidome Alterations

#### 3.2.1. Lipidome Composition in PHIV and Control Groups

**Figure 1:**
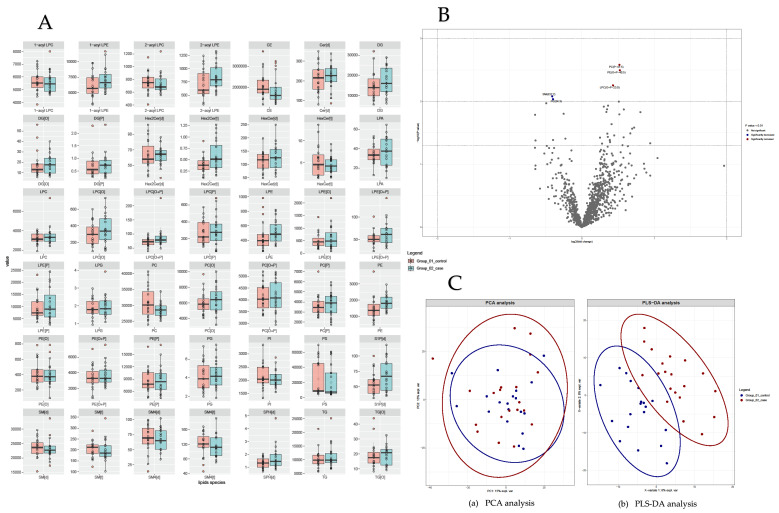
Lipidomics analyses of PHIV children compared to controls. (**A**) Boxplot of lipid concentration of key lipids identified. (**B**) Volcano plot depicting lipidomics data of 50 most changed lipid species between PHIV adolescents and controls. Significance cut off is shown in legend of volcano plot. *Y*-axis plots significance level. Three horizontal dotted lines from top to bottom indicate *p* values of <0.05, 0.01 and 0.001, respectively. Two vertical dotted lines indicate log2 (fold change) of −2 and 2. (**C**) Comparison between PHIV and control participants’ lipid composition with principal component analysis ((a) PCA) and partial least square regression discriminant analysis ((b) PLS-DA), combined to identify multidimensional direction in metabolite space explaining maximum variance between different groups. PHIV cases are indicated in red and controls are depicted in blue. Important prediction variables compared PHIV participants and controls based on DAG, CE and LPC.

The lipidomic analysis yielded 1082 unique lipid species, as shown in Figure 1. We observed no significant difference between the overall lipid composition and distribution in PHIV participants compared to controls. Specific individual lipid species showed significant alterations; (P-41:5), PE(O+P-40:5) and LPC(O+P-23:0) were significantly higher and SM(d) 32:2 species was significantly lower in PHIV participants compared to controls (*p* < 0.01), as shown in Figure 1A,B. The findings from both methods were used to represent overall lipidomic alterations. Additionally, the capacity to distinctly display significant lipid composition differences between PHIV participants and controls is represented in a PCA plot (Figure 1C). Combined PLS-DA to identify multidimensional directions within the metabolite space that account for maximum variance between groups showed no substantial significant variance between groups (Figure 1C).

#### 3.2.2. Lp(a) Increase and Various cART Regimes in Relation to Lipidomic Profiles

**Figure 2:**
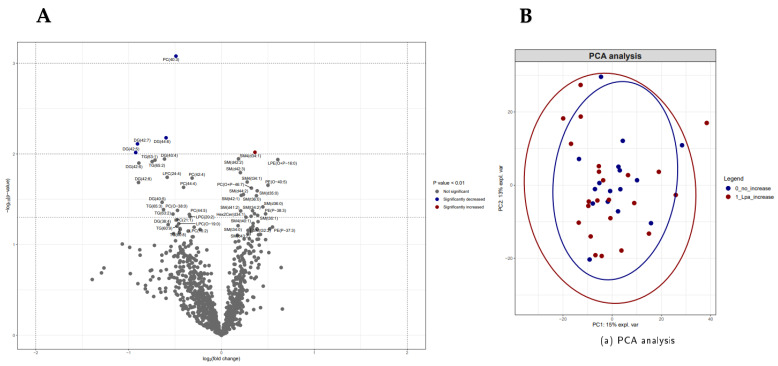
Lipidomics analyses of participants with increased Lp(a) levels ≥30 mg/dL compared to non-increased Lp(a) levels. (**A**) Volcano plot depicting lipidomics data of 50 most changed lipid species between two groups. Significance cut off is shown in legend of volcano plot. *Y*-axis plots significance level. Three horizontal dotted lines from top to bottom indicate *p* values of <0.05, 0.01 and 0.001, respectively. Two vertical dotted lines indicate log2 (fold change) of −2 and 2. (**B**) Comparison between groups of lipid composition with principal component analysis (PCA). Important prediction variables compared increased Lp(a) participants (red) and non-increased Lp(a) participants (blue) based on DAG, CE and LPC.

**Figure 3:**
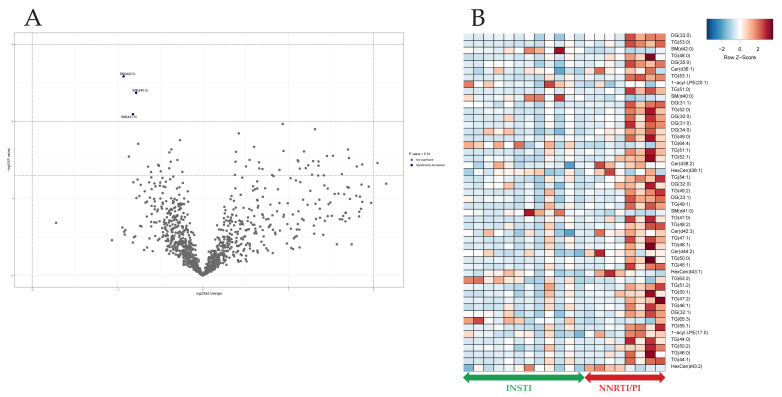
Lipidomics analyses of PHIV participants with either INSTI-based cART regimens or NNRTI/PI-based cART regimens. (**A**) Volcano plot depicting lipidomic data of 50 most changed lipid species between two groups. Significance cut off is shown in legend of volcano plot. *Y*-axis plots significance level. Three horizontal dotted lines from top to bottom indicate *p* values of <0.05, 0.01 and 0.001, respectively. Two vertical dotted lines indicate log2 (fold change) of −2 and 2. (**B**) Heatmap of 50 most important lipids based on *t*-test *p*-values across groups. Lipid species (*y*-axis) and abundance difference is shown in color (red: higher in NNRTI/PI; white: no difference; blue: lower in NNRTI/PI).

The lipidomic data of participants (PHIV as well as controls) with increased Lp(a) levels ≥30 mg/dL were compared to those participants with non-increased Lp(a) levels, as shown in Figure 2. We observed no significant difference in the overall lipid composition between the groups (Figure 2B). Individual lipid species, PC(40:3) (*p* < 0.01) and DG(42:7, 44:6 and 42:53) (*p* < 0.01), were significantly lower in participants with elevated Lp(a) levels (Figure 2A). The individual lipid species SM(d-34:1) was significantly higher in participants with elevated Lp(a) levels (*p* < 0.01). Figure 3 illustrates the lipidome composition of PHIV participants with NNRTI/PI-based regimens compared to INSTI-based cART regimens. The heatmap shows the top 50 altered lipid species based on an uncorrected p-value with visually observed alterations in individuals with NNRTI/PI-based cART regimens compared to INSTI-based cART regimens. This observed difference in species with short-chain fatty acids is not observed in all individuals with NNRTI/PI-based regimens (Figure 3B). Most lipids did not show significant differences in the univariate analysis, and only individual lipid species SM(d-40:0, d-41:0 and d-42:0) (*p* < 0.01) were significantly lower for NNRTI/PI-based cART regimes (Figure 3A). The findings from both methods were used to represent the overall lipidomic alterations.

## 4. Discussion

In this cross-sectional pilot study involving long-term cART-treated PHIV children and adolescents compared to matched HIV-negative controls, we uniquely investigated the plasma lipidome between the groups. We observed no significant difference in the lipid composition or lipidomic profiles between PHIV participants and controls. Moreover, we did not observe alterations in the lipidome when assessing elevated Lp(a) levels between the groups, a marker for cardiovascular risk. We visually observed a difference in the lipid species of PHIV participants that used NNRTI- or PI-based cART regimens compared to those who used more recent INSTI-based cART regimens; however, lipidomic profiles did not significantly differ between groups and the causality of this observation remains undetermined. In the PHIV group compared to the control group, specific lipid species within LPC, PE and PC were significantly higher. In participants with elevated Lp(a) levels, specific lipid species within PC and DAG were significantly lower.

Our study shows similar lipid composition and lipidomic profiles in PHIV children and adolescents as compared to matched controls. This is in contrast to a Ugandan study reporting increased concentrations of several lipid classes of CE, LPC, PC and SM in cART-treated and virally suppressed PHIV children between 10 and 18 years of age compared to HIV-negative controls [33]. A possible explanation for the discrepancy in findings is the different cART regimens. In our study, 55% of PHIV children were treated with INSTI, while in the only other study on PHIV participants’ lipidome, most children used NNRTI-based regimens such as efavirenz, 12/20 (60%), and nevirapine, 2/20 (10%), or PI-based regimens, 6/20 (30%) [35]. Although reports on lipidome alterations between cART regimens are scarce, it is suggested that PIs and even specific NNRTIs could alter plasma lipid profiles [19,20,21,22,42,43]. A randomized open-label trial observed reduced inflammatory lipid species when switching from an efavirenz (NNRTI)-based cART regimen to a rilpivirine (NNRTI)-based cART regimen in HIV-infected adults; therefore, specific agents from a similar class could have an influence on the plasma lipidome [44]. The possible effect of different cART treatment regimens on the plasma lipidome and CVD risk remains an area of interest in order to develop and improve future diagnostic and treatment strategies [20,22,32].

The effect of treatment on lipid composition could also explain the observed differences between the results of our study and those in adults. In HIV-infected adults, altered plasma lipidomes and associations between several lipid classes DAG, PI, PG, TAG, CER, PE, PLE, CE and CVD and metabolic syndrome risk are reported.

Specific DAG and TAG lipid classes are reported to be most associated with CVD complications [33,42]. LPC levels are reported to be increased in HIV-infected adults on cART. LPCs containing saturated fatty acids are considered pro-inflammatory in contrast to polyunsaturated fatty acids that have anti-inflammatory properties [45,46]. Conventional cardiovascular risk assessment tools often underestimate risks in HIV-infected individuals, and therefore, lipidomic analysis may offer promise in enhancing CVD risk profiling and CVD prevention treatment [30]. The PI-related risk for dyslipidemia widely varies among different agents [19,21]. Ritonavir is reported to have the most adverse impact on lipid profiles, while new agents like darunavir affect cholesterol levels to a lesser extent than older agents such as ritonavir [47]. The pathophysiology behind cART-associated dyslipidemia is a decreased uptake of fatty acids and fatty oxidation by hindering very-low-density lipoprotein (VLDL) clearance [48]. Efavirenz causes greater lipid disturbances (the occurrence of dyslipidemia and increased total cholesterol levels) compared to other ARTs [32], possibly due to interference with mitochondrial functioning, thereby reducing lipogenic factors and boosting cholesterol production [49]. Given these distinct mechanisms, different antiretroviral agents possibly affect the lipidome in various ways. A possible explanation for the difference in lipidome alterations in our study compared to HIV-infected adults might be that alterations in lipid profiles and metabolic risk only develop while aging in adulthood.

Secondly, other factors influencing the plasma lipidome could explain the discrepancy in the findings between our study and those from the Ugandan study such as a Mediterranean diet, fasting versus non-fasting samples, smoking or substance use [25,50]. Furthermore, the adolescents in this study did not use any other antibiotic medication in contrast to the PHIV children in Uganda. In this study, children used cotrimoxazole prophylaxis, with in vivo evidence indicating dysbiotic microbiotic profiles in those on antibiotic prophylaxis compared to controls [51]. Moreover, a compromised gut barrier function, possibly due to HIV infection, could contribute to heightened plasma levels of microbial products. This can adversely impact monocytes and impair HDL-mediated reverse cholesterol transport, potentially blocking ATP transporter A1 (ABCA-1)-mediated cholesterol efflux, which could impact the plasma lipidome and CVD risk [52].

Lastly, we did observe a visual trend with differences in short chain length TAGs and DAGs between PHIV participants that used PI- or NNRTI-based cART regimens compared to those who used more recent INSTI-based cART regimens. These results could not be explained by particular individual differences, such as variances in fasting criteria or cholesterol levels. Nevertheless, specific dietary or other variations that might impact our findings cannot be excluded, and hence, the significance and causality of this observation remain uncertain.

The present study has several limitations. The sample size of our study is small which may have affected the statistical power, especially in the subanalysis of different cART regimens. Secondly, the cross-sectional and exploratory design of the project does not allow for implications on causality. Furthermore, due to the small sample size, we could not correct for dietary differences, smoking, diabetes and other CVD-related comorbidities that are known to be of importance to plasma lipidomes [25,50]. The strengths of this study include a thoroughly matched control group, matched for age, sex, ethnicity and SES. With similar baseline characteristics, the likelihood of genetic or ethnic differences being accountable for our findings is reduced. Furthermore, the thorough analysis involved systematic lipid profiling utilizing quantitative lipidomics, with the quantification of over 900 lipid species.

For future considerations, it is important to evaluate the implications of our findings on the plasma lipidome and possible future CVD risk in PHIV adolescents. Our study showed similar plasma lipidomic profiles between long-term cART-treated PHIV adolescents and matched controls. However, specific cART regimens may increase levels of certain lipid species or alter the plasma lipidome, as previously described in the literature and evidenced by the observed influence on chain length differences in our study [32,43,44]. Future research with larger sample sizes is warranted to confirm these findings and better understand the long-term impact of cART on lipid levels and CVD risk in PHIV individuals transitioning into adulthood.

## 5. Conclusions

In conclusion, our study shows similar lipidomic profiles between long-term cART-treated PHIV adolescents and matched controls. Our study suggests that the plasma lipidome composition is similar when comparing matched controls to cART-treated PHIV adolescents. Specific cART regimens seem to affect lipid species with a short chain length in the plasma lipidome of PHIV adolescents and therefore induce a potential CVD risk. However, the sample size was too small to detect significant changes or establish causality and to draw firm conclusions.

Further longitudinal studies are warranted to determine the long-term differences in the plasma lipidome and the effect of different cART regimens on PHIV children and adolescents growing up into adulthood with increased CVD risk. Alterations in the plasma lipidome could provide further understanding of the effect of cART on lipid composition and could point to possible diagnostics or treatment relevant for HIV-associated CVD risk.

## Figures and Tables

**Table 1:** Participants’ characteristics.

		PHIV (n = 20)		CONTROLS (n = 20)	
	**n**		**n**		***p***
Age (years)	20	17.5 (15.5–20.7)	20	16.5 (15.7–19.8)	0.191 ^Y^
Female sex	20	9 (45%)	20	13 (65%)	0.366 ^Z^
Ethnic origin					0.999 ^Z^
Black	20	16 (80%)	20	17 (85%)	
Other ^1^		4 (20%)		3 (15%)	
Height (m)	19	1.66 (1.57–1.74)	20	1.68 (1.63–1.77)	0.278 ^Y^
Weight (kg)	19	56 (49–70)	20	64 (56–78)	0.084 ^Y^
BMI (kg/m^2^)	19	20.4 (19.2–22.3)	20	22.8 (19.8–26.3)	0.134 ^Y^
Overweight (BMI ≥ 25 kg/m^2^)	19	2 (11%)	20	4 (20%)	0.432 ^Z^
Systolic BP (mmHg) Diastolic BP (mmHg)	19 19	125 (115–133) 67 (59–76)	18	119 (114–124) 64 (59–74)	0.484 ^Y^ 0.843 ^Y^
Smoking	19 19	6 (30%)	19 19	5 (26%)	0.904 ^Z^
Lipids					
Total cholesterol (mmol/L)		3.89 (3.51–4.32)		4.31 (3.83–4.56)	0.158 ^Y^
LDL (mmol/L)		2.10 (1.75–2.67)		2.33 (2.08–2.55)	0.222 ^Y^
HDL (mmol/L)		1.33 (1.24–1.67)		1.61 (1.23–1.81)	0.5 ^Y^
Triglycerides (mmol/L)		0.83 (0.47–1.15)		0.74 (0.42–0.68)	0.259 ^Y^
Age at HIV diagnosis (years)	20	1.7 (0.8–4.2)			
CDC					
NA		8 (38%)			
B		8 (38%)			
C		4 (24%)			
Nadir CD4^+^ T-cell *Z* score (cells/µL)	19	0.82 (0.61)			
Zenith HIV viral load (log_10_ copies/mL)	18	5.5 (4.9–5.8)			
Age at cART initiation (years)	18	2.5 (1.2–6.0)			
Duration of cART (years)	18	14.9 (9.5–19.6)			
Undetectable viral load at assessment (<40 copies/mL)	20	18 (90%)			
cART regimen, No. (%) Backbone + INSTI Backbone + NNRTI Backbone + PI	20	11 (55%) 5 (25%) 4 (20%)			

Values are either reported as the median (with the interquartile range) or the number (with the percentage No%); ^1^: Caucasian or Asian; ^Y^ = Mann–Whitney U test; ^Z^ = Fisher’s exact test; CD4+ T cell Z score is age-adjusted. Abbreviations: BP = blood pressure; BMI = body mass index; cART = combination antiretroviral therapy; CDC = Center for Disease Control & Prevention; NA = no or minimal symptoms; B = moderate symptoms; C = brain AIDS; HIV = human immunodeficiency virus; kg = kilogram; l = liter; m = meter; INSTI = integrase strand transfer inhibitor; IQR = interquartile range; HDL = high-density lipoprotein; LDL = low-density lipoprotein; NNRTI = non-nucleoside reverse transcriptase inhibitor; NRTI = nucleoside transcriptase inhibitor; PI = protease inhibitor; backbone consisted of a combination of two NRTIs or one if combined with NNRTI.

## Data Availability

The data cannot be shared publicly because the data contain (potentially) sensitive patient information. The data are available (in anonymous form) upon request to the corresponding author.
